# Impact of electrode selection on modeling tDCS in the aging brain

**DOI:** 10.3389/fnhum.2023.1274114

**Published:** 2023-11-24

**Authors:** Aprinda Indahlastari, Ayden L. Dunn, Samantha Pedersen, Jessica N. Kraft, Shizu Someya, Alejandro Albizu, Adam J. Woods

**Affiliations:** ^1^Center for Cognitive Aging and Memory, McKnight Brain Institute, University of Florida, Gainesville, FL, United States; ^2^Department of Clinical and Health Psychology, College of Public Health and Health Professions, University of Florida, Gainesville, FL, United States; ^3^Department of Neuroscience, College of Medicine, University of Florida, Gainesville, FL, United States

**Keywords:** tDCS, electrodes, computational model, finite element method, cognitive aging, non-invasive brain stimulation

## Abstract

**Background:**

Person-specific computational models can estimate transcranial direct current stimulation (tDCS) current dose delivered to the brain and predict treatment response. Artificially created electrode models derived from virtual 10–20 EEG measurements are typically included in these models as current injection and removal sites. The present study directly compares current flow models generated via artificially placed electrodes (“artificial” electrode models) against those generated using real electrodes acquired from structural MRI scans (“real” electrode models) of older adults.

**Methods:**

A total of 16 individualized head models were derived from cognitively healthy older adults (mean age = 71.8 years) who participated in an in-scanner tDCS study with an F3-F4 montage. Visible tDCS electrodes captured within the MRI scans were segmented to create the “real” electrode model. In contrast, the “artificial” electrodes were generated in ROAST. Percentage differences in current density were computed in selected regions of interest (ROIs) as examples of stimulation targets within an F3-F4 montage.

**Main results:**

We found significant inverse correlations (*p* < 0.001) between median current density values and brain atrophy in both electrode pipelines with slightly larger correlations found in the artificial pipeline. The percent difference (PD) of the electrode distances between the two models predicted the median current density values computed in the ROIs, gray, and white matter, with significant correlation between electrode distance PDs and current density. The correlation between PD of the contact areas and the computed median current densities in the brain was found to be non-significant.

**Conclusions:**

This study demonstrates potential discrepancies in generated current density models using real versus artificial electrode placement when applying tDCS to an older adult cohort. Our findings strongly suggest that future tDCS clinical work should consider closely monitoring and rigorously documenting electrode location during stimulation to model tDCS montages as closely as possible to actual placement. Detailed physical electrode location data may provide more precise information and thus produce more robust tDCS modeling results.

## Introduction

The population of adults over the age of 65 has been growing at an ever-increasing rate and is expected to double by the year 2050 (Indahlastari et al., 2021b). This growth highlights the heightened need for non-invasive treatment options for cognitive decline and other brain-related health concerns. One such option is transcranial direct current stimulation (tDCS), which is a form of non-invasive brain stimulation aimed at improving brain functions and mitigating diseases in the older adult population (Brunoni et al., 2012; Woods et al., 2016). For instance, tDCS has been shown to have positive impacts in treating depression, anxiety, and Parkinson’s disease (Brunoni et al., 2013; Shiozawa et al., 2014; Alizadeh Goradel et al., 2016; Hadoush et al., 2018; Razza et al., 2020; Stein et al., 2020), as well as improving working memory performance in cognitively healthy and impaired older adults (Berryhill and Jones, 2012; Saunders et al., 2015; Gomes et al., 2019; Ciullo et al., 2021; Rodella et al., 2021). The application of tDCS involves affixing two or more electrodes, consisting of the anodes (current injection site) and the cathodes (current removal site), to specific positions on the scalp. This setup allows for the delivery of a mild electrical current (e.g., 1–2 mA) to stimulate the targeted brain regions (Indahlastari et al., 2019a,2019b). The assumption underlying tDCS mechanisms is that the delivered electrical current will reach the brain tissues, causing depolarization or hyperpolarization of neuronal membrane potentials and affecting neural plasticity (Brunoni et al., 2012).

Previous studies suggest that the outcomes of tDCS can vary among recipients and one important factor contributing to this variation is inter-individual differences in anatomy. In the aging population, this observation is particularly influenced by structural degeneration, such as brain atrophy, which occurs during the aging process. However, measuring the electrical current delivered by tDCS to the cortical region *in vivo* remains challenging. To overcome this, person-specific models are constructed using individual T1-weighted MRI structural images to estimate the distribution of current delivered by tDCS within the head. These models are often used to compute the current dose associated with treatment response (Huang et al., 2013; Windhoff et al., 2013; Indahlastari and Sadleir, 2015; Indahlastari et al., 2020). Further, age-related structural decline can affect the amount of current delivered in the brain. For example, our previous modeling study demonstrated that applying a fixed dosage across a sample of older adults resulted in varying distributions of electrical current in the brain. Fixed dosing refers to prescribing the same stimulation parameters, including current intensity and electrode montage. Specifically, older adults’ brains received less current compared to their young adult counterparts when the same dosage was applied (Indahlastari et al., 2020).

To ensure accurate model estimates, it is crucial to place the electrodes in precise stimulation locations within the models, as this significantly impacts the accuracy of field measure estimates. Typically, the electrodes in tDCS computational models are artificially created during modeling pipeline in their ideal location based on the standard 10–20 EEG system. Our previous brief report (Indahlastari et al., 2023) compared current density results generated from these artificial electrodes (artificial electrode model) to those generated by using segmented electrodes at their real location (real electrode model). Our prior findings indicated that electrical current distribution produced from using artificially placed electrodes were significantly different from those using real electrode location, with varying percent difference ranging from 6.59 to 35.54% in the brain. Differences in current densities within each tissue compartment ranged from 20.55 to 37.22%, with skin exhibit the largest discrepancy (Woods et al., 2015; Opitz et al., 2016; Indahlastari et al., 2019a; Knotkova et al., 2019).

In a prior analysis, we demonstrated that merely using the planned electrode locations produced current density models that were significantly different than those generated using the actual electrode locations (Indahlastari et al., 2023). The present paper aims to expand our prior comparison by investigating the effects of electrode properties due to significant changes between the two electrode models. To that end, the current study analyzed the differences in contact area and electrode distance that could potentially contribute to any significant changes seen between the two electrode models. In addition, this study also assessed whether each electrode pipeline would be sufficient to make predictions regarding the delivered current dose and age-related structural decline, such as brain atrophy. We hypothesize that both electrode pipelines can significantly predict the relationship between current density and brain atrophy in our older adult sample. The implication of the present study is to caution tDCS researchers about the importance of accurately representing electrode locations when modeling tDCS for the same study.

## Materials and methods

A total of sixteen cognitively healthy older adults (mean age = 71.8 years) received in-scanner tDCS during MR imaging sessions. Cognitive status was determined using the Montreal Cognitive Assessment (MoCA) and deemed healthy with a score of 20 and above. Additional details and information pertaining to participant screening and recruitment can be found in the previously published paper by Nissim et al. (2019). Individual T1-weighted images were converted to head models using a combination of manual and automatic processes. All models were executed using ROAST with minimal modification to simulate an end-to-end automatic tDCS modeling pipeline. Auto-segmented tissues were further corrected manually to ensure that any discrepancy in the generated results were only sourced from the different electrode models instead of from inaccurate tissue assignments. Individual segmented volumes in both electrode pipelines were identical to isolate the differences in current densities as originating solely from different electrode models. Further details of the study methods are outlined in the following subsections.

### Imaging parameters and tDCS set-up

T1-weighted images relevant to model construction were generated using the MPRAGE sequence in a 3T Philips Achieva MRI Scanner (FOV = 240 × 240 × 170 mm, voxel size = 1 mm^3^, TR = 7 ms, TE = 3.2 ms, flip angle = 8°). Conventional tDCS was applied inside the MR scanner using a NeuroConn DC-Stimulator for 12 min during the active condition and for 30 s during the sham condition, with an additional 30 s of ramp up/down. Prior to the imaging session, two 5 × 7 cm rubber pad electrodes with a layer of Ten20 conductive paste (∼5 mm thickness) were affixed to each participant at the F3 (cathode) and F4 (anode) locations following the standard 10–20 EEG System outside of the MR scanner (Nissim et al., 2019). A 32-channel head coil was then placed on participant’s head after the electrodes were affixed to the head, prior to the start of the MRI scan.

### Head model construction

**FIGURE 1:**
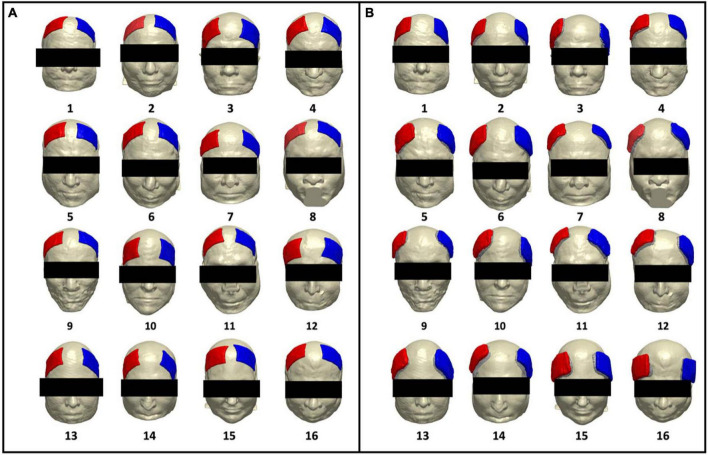
Renderings of artificial and real electrode models on each participant. Participant number is denoted with numeric values below each head model. Electrode pad (red) and paste (green) are rendered in each participant for **(A)** artificially generated electrodes (artificial electrode models) and **(B)** segmented electrodes from acquired T1-weighted images (real electrode models), depicting the location of the F3 (cathode) and F4 (anode) electrode montage.

Acquired T1-weighted images were converted into head models using a combination of automatic and manual processes. Each head model was segmented into six tissue types: white matter (including optic nerve and spinal cord), gray matter, cerebrospinal fluid (CSF), bone, air, and skin. The number of tissue types were selected to match the default setting of the ROAST 3.0 pipeline (Huang et al., 2019). All automatically segmented tissues of white matter, gray matter, CSF, bone, and air were manually corrected in Simpleware ScanIP module (Synopsys Ltd., CA, USA) with a reference to an atlas following our published methods (Indahlastari et al., 2021a). The remaining tissue regions outside of the five segmented masks were categorized as skin. Since the head coil was placed over the electrodes at the time of stimulation, an imprint of electrode paste at each electrode location was visible on the scalp/skin mask (Figure 1, Appendix B, in the Supplementary material). Fully segmented head volumes were then exported to ROAST for the remaining modeling steps. Details of each modeled electrode’s construction are described in the next subsections.

#### Artificial electrode model

The artificial electrode pad and gel were generated by executing the default ROAST pipeline. Each electrode and gel were placed at the F3 (cathode) and F4 (anode) locations on our segmented volumes. Since the auto-segmented skin had imprints of actual electrodes, the auto-generated gel in ROAST did not cover the entire electrode and scalp interface area, leaving a gap between generated electrodes and the scalp (skin mask). Prior to volume meshing, the missing gel regions were manually added in Simpleware to ensure there is no gap between the electrodes and the scalp.

#### Real electrode model

Since tDCS was performed inside the MR scanner, the electrode and paste were visible in acquired T1-weighted images for each participant, as illustrated in Supplementary Figure 2 (Appendix B, in the Supplementary material). Each electrode pad and paste were manually segmented from the T1 images in Simpleware and exported as binary masks to run the real electrode models. These segmented electrode pad and paste volumes then replaced the automatically generated electrode pad and gel (artificial electrodes) within ROAST.

### Finite element simulation

The remaining portions of the modeling pipeline, including volume meshing and solution generation, for both electrode models were executed using ROAST 3.0 within Matlab R2020a (Mathworks, Natick, MA). The input command in ROAST assigned F4 as the anode electrode and F3 as the cathode electrode with a 70 × 50 × 3 mm pad size and 2 mA input current. In the default ROAST, the size of the gel including the thickness mimics the dimensions of the assigned pads. White matter, gray matter, CSF, bone, and skin were assigned ROAST’s default conductivity values of 0.126S/m, 0.276S/m, 1.65S/m, 0.01S/m, and 0.465S/m, respectively (Bestmann and Ward, 2017; Huang et al., 2019). An additional conductivity of 0.3178 S/m was assigned to the electrode paste (Gilad et al., 2007) within the real electrode models. The segmentation steps within ROAST were skipped to accommodate for using the corrected segmentation volumes as input. The electrode placement module of ROAST, during which artificial electrodes are placed, was skipped during real electrode model execution. Both electrode models were meshed using iso2mesh and solved with getDP within the ROAST software. The Neumann boundary condition was assigned using the open-source solver getDP, which injects current at the F4 electrode and removes it at the F3 electrode, while ensuring that there is no current leakage anywhere on the head surface. The simulation produced 32 electric field volumes, comprising 16 real models and 16 artificial models. Current density volumes were then generated by multiplying the electric field volumes with conductivity volumes of the six tissue types.

### Electrode property

In each electrode model, electrode contact area information for artificial and real electrodes were sourced from the ROAST output. The normalized electrode areas were computed by dividing each contact areas by the averaged contact areas for the anode and cathode electrodes within the artificial electrode model, and then averaging the overall anode and cathode values together. We found that the generated contact areas from ROAST default deviated from the intended 35 cm^2^ electrode size. We believe this discrepancy occurred from variations in scalp topology, which differed between individual heads. Consequently, the electrodes might not always perfectly align with the scalp or achieve a precise 35 cm^2^ coverage. Due to this inconsistency, we calculated the average for all ‘artificial’ contact areas and used this value to normalize all contact areas. Since our study aims to compare the electrode properties of the two pipelines, we chose not to use the intended contact area value (35 cm^2^) and instead use the normalized values as a metric of difference across electrodes. Electrode separation distance between the anode and cathode electrodes was computed in each model to quantify the relative location of F3 and F4 placements. These distances were calculated as linear distances (the Euclidean distance) and were computed as direct distances between the midpoints of the short electrode edges of the anode and cathode electrodes, as illustrated in Supplementary Figure 3 (Appendix C, in the Supplementary material).

### Current density

Field maps generated by ROAST using two distinct electrode paradigms were compared to discern any discrepancy in the generated results. In each participant, the generated electric field and current density values were compared between the artificial and real electrode pipelines. To demonstrate the practical considerations of the effect of electrode models on delivered current densities in the aging brain, current density volumes were further isolated in sub-regions such as the whole brain, gray and white matter, as well as subcortical regions of interest (ROIs). Three ROIs were selected to represent the typical stimulation targets for F3-F4 location, and the regions commonly found to be the first to decline in aging populations (Indahlastari et al., 2021a). The selected ROIs include the superior frontal gyrus (SFG) and the middle frontal gyrus (MFG) as an anatomical representation of the commonly functional region, the dorsolateral prefrontal cortex (DLPFC) commonly targeted by F3-F4 montage (Rajkowska and Goldman-Rakic, 1995; Desikan et al., 2006; Kikinis et al., 2010; Mai and Paxinos, 2012; Cieslik et al., 2013). In addition, we included the inferior frontal gyrus (IFG) as an alternative targeted region with this montage (DaSilva et al., 2015; Stramaccia et al., 2015; Thunberg et al., 2020). The temporal lobe was included as an example of a non-target region nearby the electrode location. All ROIs were segmented using FreeSurfer v6.0. Tissue volumes, such as gray matter, white matter, and sub-brain regions, were computed in Matlab to estimate brain atrophy level as represented by a total volume ratio for each brain region (Indahlastari et al., 2020). Median values for each electric field and current density volumes were computed for comparison between the two electrode models. The median values were used instead of the mean values as median values demonstrate measures of central tendency that are not skewed by outlier data (Laerd Statistics, 2018).

### Statistical analysis

Paired *t*-tests between the median values of current densities in artificial versus real electrode models were carried out using IBM SPSS Statistics version 27. Percent differences (PD) were computed as the absolute differences between values obtained in artificial and real electrode models relative to the real electrode models. In addition, linear regression analyses to correlate volume ratio and the computed current densities in both electrode models were also performed in SPSS. Computed current densities were then further restricted to the four selected ROIs for each electrode model and correlated with electrode properties to discern any relationship between them.

## Results

Electrode locations for artificial and real electrode models were rendered on individual head models to illustrate the generated electrode location for each electrode type. Contact areas and separation distance between anode and cathode electrodes were plotted to quantify the variation in the shape of the electrodes generated across the two models. Current density and electric field maps were also generated for each of the sixteen participants using both the artificial and real electrode models (32 volumes total). The details of these comparisons and further analyses are described in the following subsections.

### Electrode location

The artificial and real electrode models are rendered on individual participants as seen in Figure 1. This figure is a rendition from our prior brief report and is included in this paper to showcase the electrode locations from both pipelines as rendered onto individual participants. Figure 1A depicts the location of generated artificial electrodes and shows more consistency in electrode shape, distance of separation between anode and cathode, as well as placement on each head model. In contrast, Figure 1B depicts electrode placement for the real electrode models and visually demonstrates larger variations in electrode shape, separation distance, and location on the scalp. The real electrode models also demonstrated less symmetry with respect to the midline of the head between the location of the anode (F4) and cathode (F3) electrodes.

**FIGURE 2:**
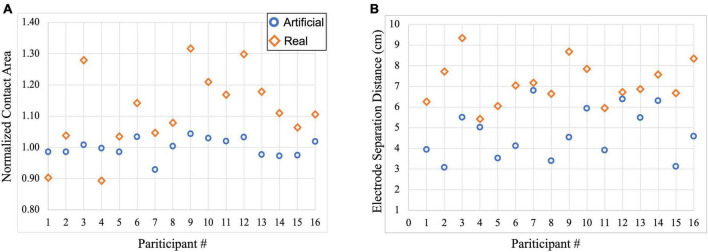
Scatterplot of electrode information for each participant within the artificial and real electrode models. Blue circles represent contact areas computed in the artificial electrode models while orange diamonds represent contact areas computed in the real electrode models. **(A)** Normalized contact areas were plotted for artificial (mean ± SD: 1.00 ± 0.03) and real (mean ± SD: 1.12 ± 0.12) electrode models, and **(B)** Electrode separation distance between the anode (F4) and cathode (F3) electrodes were computed in the artificial (mean ± SD: 4.73 ± 1.22 cm) and real (mean ± SD: 7.15 ± 1.06 cm) electrode models.

Contact areas and separation distances between electrodes were computed to quantify the variation seen in electrode shape between the two electrode models. Figure 2A illustrates the distribution of the computed normalized electrode area across participants. As expected, the blue circles representing contact areas in the artificial electrode models are more consistent in size (mean ± SD: 1.00 ± 0.03) as most of the normalized areas are closer to 1 as shown in Figure 2A. On the other hand, the real electrode models (orange diamonds) showed a larger variation of contact areas (mean ± SD: 1.12 ± 0.12). Normalized values were computed with respect to the average electrode size. Figure 2B depicts the variation in electrode separation distance between the anode (F4) and cathode (F3) electrodes in each model. In addition, overall larger separation distances were observed for the real electrode models (mean ± SD: 7.15 ± 1.06 cm), especially for Subject 3, 9, and 16, compared to the artificial electrode models (mean ± SD: 4.73 ± 1.22 cm).

### Field maps

**FIGURE 3:**
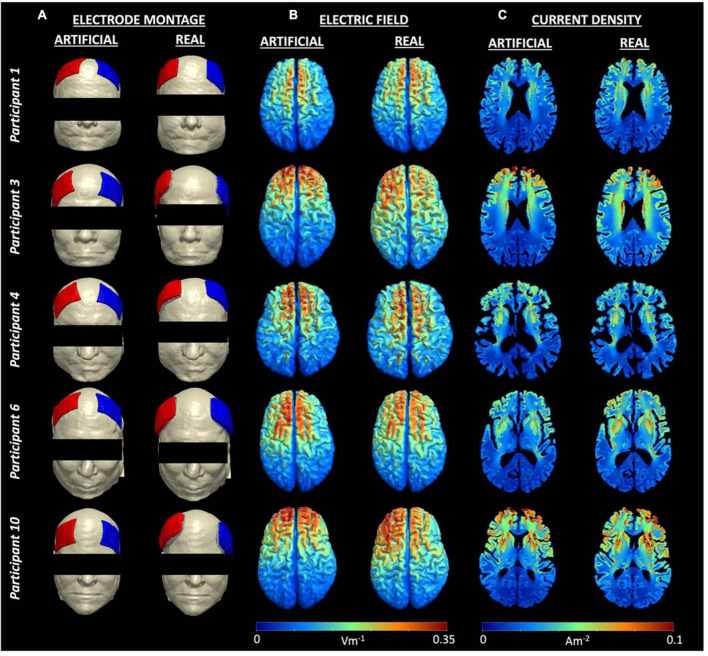
Electrode montage and field measures for five example participants. **(A)** Electrode montage for each person showing F4 as the anode (blue) and F3 as the cathode (red), **(B)** Individual electric fields, and **(C)** Individual current density volumes generated using the artificial (left) and real (right) electrode models for participant 1, 3, 4, 6, and 10. Visualizations of all electrode montages are included in Figure 1.

The generated field measures in the form of electric field and current density volumes isolated to the brain (gray and white matter) regions were plotted in each head model. Figure 3 shows the electric field and current density distributions of five participants as an example of the output from the artificial and real electrode pipelines. The remaining field measure visualizations can be found in our previous publication (Indahlastari et al., 2023). Overall, the electric field distribution pattern was generally similar across subjects with larger values in the regions near the electrodes and smaller values in the posterior regions, farther away from the electrodes. Both electrode models demonstrated large electric fields on the cortical surface located between the anode and cathode placement, which is consistent with prior research. In addition, a more symmetric distribution of high and low values across the brain hemispheres was seen in the artificial electrode models, which was expected given that the electrode placement for the artificial electrode models were more consistent than the real electrode models as seen in Figure 1. Visually, the variation in current densities across participants provided additional insight regarding the location of the selected slice respective to the electrodes’ placement. Participant 3 and 10, for example, exhibits larger values in the frontal cortex within the artificial models, wherein the anode and cathode were placed closer together compared to the real models. Variations in anatomical structures across participants also contributed to the high and low values of current densities. The lateral ventricles appeared “larger” in participant 1, 4 compared to participant 3, 10, indicated a larger degree of brain atrophy. Therefore, the overall range of current densities was smaller (cooler color) for participant 1, 4 than participant 3, 10.

**FIGURE 4:**
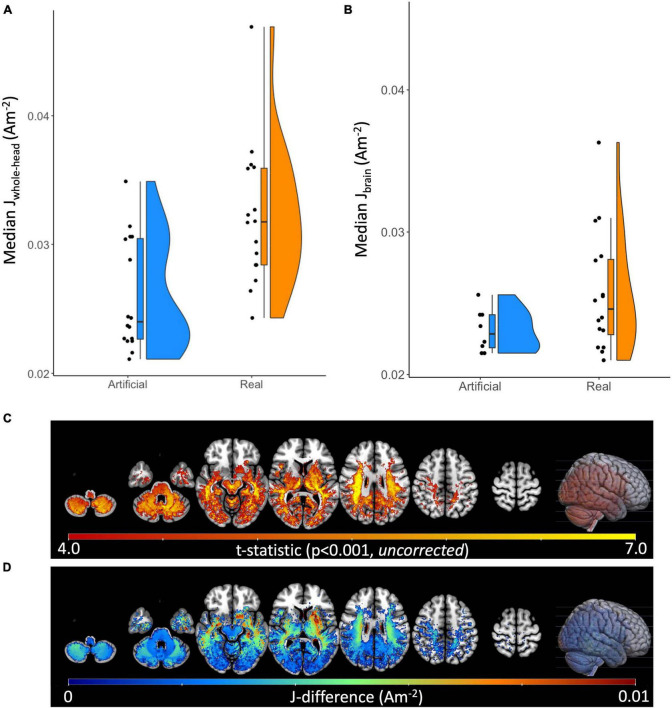
Raincloud plots, t-statistic maps, and current density difference maps of artificial versus real electrode models. Raincloud plots depicted as a combination of boxplot, jittered point, and halved violin of computed median current densities for both electrode models. Performed paired *t*-test were found significant (*p* < 0.001) for **(A)** Median J_whole–head_ and **(B)** Median J_brain_ between the artificial and real electrode models. **(C)** The t-statistic map for the Real > Artificial contrast at *p* < 0.001 (*uncorrected*), plotted on the MNI152 template. **(D)** The Real minus Artificial current density difference map (Am^– 2^) within regions of statistically significant difference between conditions (*p* < 0.001, *uncorrected*), plotted on the MNI152 template.

Quantitatively, median current densities of the real electrode models were significantly larger (*p* < 0.001), on average (mean ± SD: 0.026 ± 0.004A/m^2^), compared to those of the artificial electrode models (mean ± SD: 0.02 ± 0.002A/m^2^) within the brain. Computed median current densities were also significantly larger (*p* < 0.001) for the four ROIs (mean ± SD_*SFG*_: 0.050 ± 0.006A/m^2^, mean ± SD_*MFG*_: 0.049 ± 0.007A/m^2^, mean ± SD_*IFG*_: 0.044 ± 0.009A/m^2^, mean ± SD_*TMP*_: 0.028 ± 0.005A/m^2^). The percent difference (PD) between the median current density (J) values of the artificial and real electrode models within the brain region ranged 6.59–35.54%, as shown in Table 1. In addition to the restricted brain region, PDs of the whole head were also computed to depict any differences of J between the two electrode types across the entire head volume. PDs ranged 0–37.33% for J values within the whole-head region. Paired *t*-tests were performed between the ideal and actual median datasets for J_*whole–head*_, as well as J_*brain*_. There was a significant difference (*p* < 0.001) found between the population means of the ideal and actual datasets for J_*whole–head*_, indicating a significant difference in current density values produced from the two separate modeling pipelines. The same difference (*p* < 0.001) was observed in the *t*-test comparing the ideal and actual median values for J_*brain*_. The *t*-test visualization, including the raincloud plots, t-statistic maps, and J-difference maps, is illustrated in Figure 4. Table 2 describes the *t*-test results in the selected regions of interest including the superior frontal gyrus, middle frontal gyrus, inferior frontal gyrus, and temporal lobe.

**TABLE 1:** Percent difference between median current densities in artificial versus real electrode models.

Participant	Median J whole-head (A/m^2^)		PD (%)	Median J brain (A/m^2^)		PD (%)
	Artificial	Real		Artificial	Real	
1	0.0227	0.0284	20.07	0.0191	0.0219	12.79
2	0.0225	0.0359	37.33	0.0215	0.0308	30.19
3	0.0349	0.0469	25.59	0.0256	0.0363	29.48
4	0.0243	0.0243	0.00	0.0206	0.0232	11.21
5	0.0211	0.0272	22.43	0.0215	0.0255	15.69
6	0.0244	0.0323	24.46	0.0234	0.028	16.43
7	0.0306	0.0293	4.44	0.0186	0.021	11.43
8	0.0227	0.0302	24.83	0.0186	0.0231	19.48
9	0.0236	0.0317	25.55	0.0191	0.0252	24.21
10	0.0314	0.0372	15.59	0.0242	0.031	21.94
11	0.0225	0.0264	14.77	0.0186	0.0219	15.07
12	0.0288	0.0284	1.41	0.022	0.0256	14.06
13	0.0306	0.0362	15.47	0.0223	0.0238	6.30
14	0.0304	0.0318	4.40	0.0189	0.0216	12.50
15	0.0237	0.036	34.17	0.0242	0.0283	14.49
16	0.0216	0.0327	33.94	0.0204	0.024	15.00

**TABLE 2:** Difference in current density, *t*-statistic, and *p*-value between the two electrode models for selected ROIs.

Region of Interest (ROI)	ΔJ_brain_ (Am^–2^)	t-statistic	*p*-value
Superior Frontal Gyrus (SFG)	0.00392	4.89521	0.00019*
Middle Frontal Gyrus (MFG)	0.00245	4.04387	0.03156*
Inferior Frontal Gyrus (IFG)	0.00306	2.94485	0.15950
Temporal lobe	0.00551	5.17923	0.00013*

*Indicates significance at *p* < 0.05.

### Electrode property and current density

**FIGURE 5:**
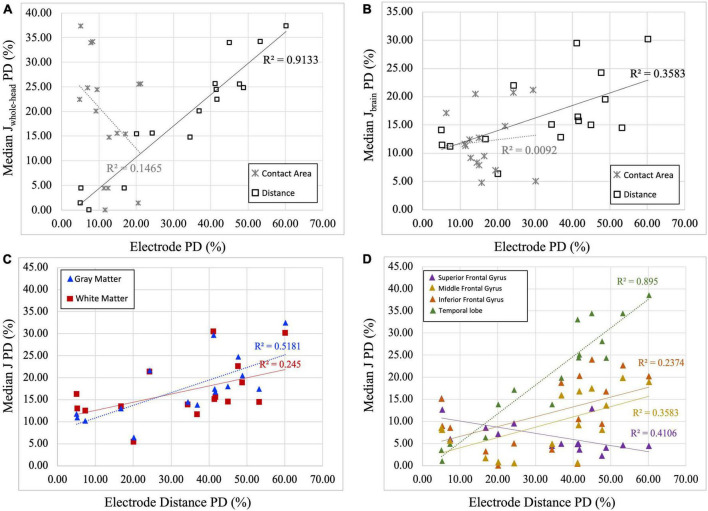
Correlation plots between current density and electrode properties. **(A)** Percent difference (PD) of median current density of the whole head (Median J_whole–head_) are correlated with electrode distance and contact area. Significant positive correlation between J and electrode distance PDs (*R*^2^ = 0.9133, *p* = 0.001) was found, while non-significant correlation was found between electrode contact area and J PDs (*R*^2^ = 0.1465, *p* = 0.143). **(B)** Percent difference (PD) of median current density of the brain region (Median J_brain_) are correlated with electrode distance and contact area. Significant positive correlation between J and electrode distance PDs (*R*^2^ = 0.3583, *p* = 0.014), while no correlation was found between electrode contact area and J PDs (*R*^2^ = 0.0092, *p* = 0.724). Therefore, only electrode distance PDs are plotted against median current densities in panels **(C,D)**. **(C)** Percent difference (PD) of median current density (J) in the gray and white matter is correlated with electrode distance PDs. Significant positive correlations between J and electrode distance are observed in gray matter regions (*R*^2^_GM_ = 0.5181, *p* = 0.02), but not in the white matter region (*R*^2^_WM_ = 0.2450, *p* = 0.051). **(D)** Percent difference (PD) of median current density (J) in selected regions of interest is correlated with electrode distance PD. Significant negative correlation between electrode distance and computed current densities was found in the superior frontal gyrus (*R*^2^_SFG_ = 0.4106, *p* = 0.007) and significant positive correlation was found in the middle frontal gyrus (*R*^2^_MFG_ = 0.3583, *p* = 0.014) and temporal lobe (*R*^2^_TMP_ = 0.8950, *p* = 0.0001). While positive, non-significant correlation was found for the inferior frontal gyrus (*R*^2^_IFG_ = 0.2374, *p* = 0.056).

Figure 5 illustrates the relationship between the computed percentage differences for current density and electrode properties (i.e., electrode distance and contact area). All percent differences reflected the absolute difference values between the artificial and real electrode models. Figures 5A, B demonstrates that the percent difference (PD) in electrode distance has a significant positive relationship with the percentage difference of current densities, both in the whole-head (*R*^2^ = 0.913, *p* = 0.001) and in the brain (*R*^2^ = 0.3583, *p* = 0.014). In contrast, contact area measures showed non-significant correlation with current densities for the whole-head and in the brain region. Since electrode distance was positively correlated with computed current densities, further correlation analyses were performed for the electrode distance PDs in various regions within the brain. Figure 5C shows significant positive correlation between electrode distance and current densities isolated in gray matter regions (*R*^2^_*GM*_ = 0.518, *p* = 0.02) and non-significant correlation for white matter regions (*R*^2^_*WM*_ = 0.245, *p* = 0.051). Figure 5D illustrates the significant negative correlation found between distance and current density PDs computed in the superior frontal gyrus (*R*^2^_*SFG*_ = 0.411, *p* = 0.007), and the significant positive correlation found in the middle frontal gyrus (*R*^2^_*MFG*_ = 0.3583, *p* = 0.014) and the temporal lobe (*R*^2^_*TMP*_ = 0.895, *p* = 0.0001), whereas a non-significant positive correlation was found for the inferior frontal gyrus (*R*^2^_*IFG*_ = 0.237, *p* = 0.056).

### Brain atrophy and current density

**FIGURE 6:**
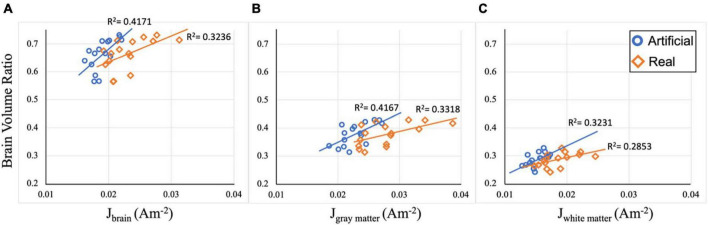
Correlation plots between current density and volume ratio. **(A)** Median current density (J) in the brain region for both electrode models are correlated with brain volume ratio. Significant positive correlation between J and brain ratio is observed with *R*^2^_brain_artificial_ = 0.4171 (*p* = 0.007) and *R*^2^_brain_real_ = 0.3236 (*p* = 0.021). **(B)** Median current density (J) in the gray matter region for both electrode models are correlated with gray matter volume ratio. Significant positive correlation between J and volume ratio is observed with *R*^2^_GM_artificial_ = 0.4167 (*p* = 0.007), *R*^2^_GM_real_ = 0.3318 (*p* = 0.02). **(C)** Median current density (J) in the white matter region for both electrode models are correlated with white matter volume ratio. Significant positive correlation between J and volume ratio is observed with *R*^2^_WM_artificial_ = 0.3231 (*p* = 0.022), and *R*^2^_WM_real_ = 0.2853 (*p* = 0.033).

Computed current densities were correlated against participants’ volume ratios to assess the relationship between current density and brain atrophy in each electrode model. Figure 6 illustrates the scatter plots for current density restricted to the whole-brain, gray matter, and white matter. When considering the relationship between the brain (white matter and gray matter) and the median current density in the brain region, both electrode models showed significant positive correlation with *R*^2^ = 0.4171 (*p* = 0.007) and *R*^2^ = 0.3236 (*p* = 0.021) for artificial and ideal electrode models, respectively (Figure 6A). Current density restricted to the gray matter (GM) and white matter (WM) also shows significant positive correlation for both electrode models (Figures 6B, C with the following values: *R*^2^_*GM_artificial*_ = 0.4167 (*p* = 0.007), *R*^2^_*GM_real*_ = 0.3318 (*p* = 0.02), *R*^2^_*WM_artificial*_ = 0.3231 (*p* = 0.022), and *R*^2^_*WM_real*_ = 0.2853 (*p* = 0.033). Overall, the computed correlation coefficients (R^2^) are, on average, 7% larger in the artificial models compared to the real models.

## Discussion

The present paper compares current density volumes generated using artificial electrode models versus those generated using real electrode models in older adults. This study expanded analyses from our previous publication that directly compared field maps generated from the two types of electrode models in order to estimate the current density distribution in conventional tDCS in relation to individual levels of brain atrophy. FEM was used to solve the Laplace equation with an applied current of 2mA total, injected at the F4 (anode) site and removed at the F3 (cathode) electrode location. Sixteen segmented head volumes consisting of six tissue types (bone, skin, air, CSF, white and gray matter) were derived from T1-weighted images of older adults. Each participant had identical segmented tissue volumes for each electrode model (artificial and real) to ensure that any differences observed from the generated field measures were solely attributable to the differences in electrode generation methods. While all models attempted to mimic F3 and F4 electrode locations, variations in the placement of electrodes at F3 and F4 was observed across participants. Contact areas, on average, were found to be more consistent in the artificial electrode models (mean ± SD: 1.00 ± 0.03) while the real electrode models demonstrated a slightly larger variation of contact areas (mean ± SD: 1.12 ± 0.12). Significant differences (*p* < 0.001) in the computed current densities in the brain and ROIs were found between the two electrode models. Contact areas showed a non-significant correlation to the percent difference in current densities, while electrode separation distance was significantly (*p* = 0.001) correlated to differences in current densities between the two electrode types.

### Field measure comparison

Significant differences (*p* < 0.001) in current density values generated by the artificial and real electrode pipelines provide insight into the importance of incorporating correct electrode geometry to improve the accuracy of field measure predictions in tDCS. When examining the entire brain volume, the significant difference in current density volumes, as illustrated in Figure 4, excluded the frontal brain region. This pattern was primarily due to the low current density values that accounted for the observed difference. A sub-region analysis, conducted by performing a *t*-test on ROIs within the frontal region, revealed significant differences in current densities between the two electrode models in both the superior frontal gyrus and the middle frontal gyrus (Table 2). In addition, the field maps of the artificial electrode model consistently showed lower median values compared to the real electrode model in the brain, and this was also observed in the majority of the whole head. These lower current density values may indicate that computational models underestimate the current density values in regions of interest for tDCS recipients, especially those with larger degrees of brain atrophy. Therefore, generated models using artificial placement of electrodes without any information regarding the physical location of the electrodes at the time of stimulation should be considered with caution, as they may not accurately depict the current dose delivered to desired brain tissue. To that end, obtaining or documenting the physical electrode placement information may be necessary to improve upon the accuracy of generated current flow models mimicking tDCS with conventional electrode configuration (large pads, typically using 10–20 EEG locations).

Electrode location is essential in dictating the electric field distribution within person-specific current flow models. Within conventional tDCS, previous modeling studies demonstrate that the largest electric field values are typically located somewhere in between the anode and cathode electrodes rather than directly underneath each electrode (Indahlastari et al., 2020). Our findings support this statement, where the pattern of the electric fields (Figure 3) is highly dependent on the electrode location for each person. For instance, participant 10 exhibits a less symmetric bilateral distribution of field measure in the real electrode model compared to the artificial electrode model. Based on the electrode location provided in Figure 3A, the anode and cathode location for the real model in participant 10 are both shifted toward the left ear and thus reflected the asymmetric electric field of Figure 3B with a larger field appearing in the left hemisphere. This observation directly supports the notion that the resulting field distributions from current density models are highly dependent on electrode location.

### Electrode impact on current density

When considering electrode properties, greater discrepancies in the delivered current density were largely contributed to electrode separation distances as compared to measured contact areas. Contact area refers to the electrode surface area interfacing with the scalp. The contact area represents the sum of the current density, which is the input current divided by the electrode surface area, and it directly affects the distribution of current. For instance, with a 35 cm^2^ contact area and a 2mA input current, the total current density would be 2mA divided by 35 cm^2^, which equals 0.057Am^–2^. This value will increase if the contact area is smaller for the same input current. Hence, changes in contact areas can influence the amount of current entering and exiting the head through the electrodes and can affect the brain regions it reaches. Typically, a larger contact area distributes the current over a broader surface, leading to a lower concentration of applied current density to the head. Smaller contact areas, such as in HD-tDCS, results in more focal and intense applications of current density and thus can be used to target brain regions near the electrodes (Dmochowski et al., 2011; Datta et al., 2012; Alam et al., 2016; Mikkonen et al., 2020). On the other hand, electrode separation distance represents the location of the anode with respect to the cathode electrode. A pair of electrodes that are placed closely together will experience shunting, wherein most of the electrical current passes through the shortest distance between the two electrodes that provides the current pathway of least resistance, rather than traveling from the anode, through the scalp and underlying head tissues, and toward the cathode (Thomas et al., 2017; Mahdavi and Towhidkhah, 2018). In prior research, the distance between F3 and F4 location can be approximated by computing the Euclidean distance (Indahlastari et al., 2019a). While the differences between the artificial and real electrode models in both their measures of contact areas and electrode distance were both significant, electrode distance showed a significant positive correlation when compared to the percent differences in current density in the brain and in selected ROIs. Therefore, in our study, this observation implies that the separation distance between the anode and cathode electrodes has a greater impact than contact areas on generated current densities. However, this observation may not be generalizable to other studies as prior research has shown that greater difference in contact areas, particularly the imbalance of anode and cathode electrode size, can cause significant changes in the current density delivered to the brain (Bikson et al., 2010; Faria et al., 2011; Saturnino et al., 2015).

### Brain atrophy and individual variability

Computed brain volume ratio, an indicator of the degree of brain atrophy in older adults, was positively correlated with the generated current density, irrespective of the electrode pipeline used. Brain atrophy is defined as the degeneration of tissue that leads to structural changes and neuron organization in the brain (white and gray matter) that can naturally occur with age (Indahlastari and Woods, 2019). In this study, brain atrophy was represented as the inverse of brain volume ratio, calculated as the sum of gray and white matter volumes divided by the intracranial volume (CSF, white and gray matter). Median current densities in white and gray matter from both the artificial and real electrode pipelines were positively correlated to the computed brain volume ratio, which indicated a trend of larger current density paired with larger volume ratio. This implies that increasing brain atrophy resulted in less current reaching the brain contributed by the thickness of CSF layer surrounding the brain, which agrees with prior research in this area (Thomas et al., 2017; Mahdavi and Towhidkhah, 2018; Indahlastari et al., 2020). Further, a higher correlation coefficient was observed in the artificial electrode models compared to the real electrode models. This provides insight into the role of the electrode in relation to atrophy sites. The placement of the artificial electrodes was more consistent and exhibited less variance in current density values. As a result, they delivered current to areas with thicker CSF, leading to a reduced amount of current delivered within the brain. This resulted in a slightly stronger correlation between current density and brain volumes. Additionally, while the two correlation coefficients from each electrode model were not identical, they both demonstrate a significant correlation to changes in brain volumes. Therefore, this finding serves practical applications of current density modeling in older adults and demonstrates how the electrode model discrepancy can affect results. Within this study population, since both electrode models produced significant correlations to computed brain volume ratio, either electrode pipeline can be used to predict the effects of brain atrophy on the delivery of current densities. However, this observation will need to be tested in a larger sample and with different population types, such as young adults or impaired older adults.

Variations in the distribution of current density across participants exists within both the artificial and real electrode model comparisons, highlighting the importance of constructing person-specific models. Prior research demonstrates that variability in current density and electric field distribution is mainly attributed to inter-individual anatomical variation (Datta et al., 2012; Laakso et al., 2015). Similarly, variability exists across participants in our generated models as shown in Figure 3. For instance, when comparing the lateral ventricles between participant 4 and 6 in Figure 3C, participant 4 shows larger lateral ventricles, indicative of greater brain atrophy. Therefore, participant 4 might not receive as much current to their brain, given that current would localize mostly within the CSF due to its higher conductivity. This is verified by comparing the median brain current density values between these two participants with smaller median values reported for participant 4 (0.0185A/m^2^ for artificial, 0.0208 A/m^2^ for real electrode models) than those reported for participant 6 (0.0199 A/m^2^ for artificial, 0.0238 A/m^2^ for real electrode models). This observation also agrees with prior modeling studies that compute current density in association with degree of brain atrophy (Thomas et al., 2017; Mahdavi and Towhidkhah, 2018; Indahlastari et al., 2020). Therefore, constructing individual head models remain crucial to ensure model accuracy and interpretation of findings.

### Model implication

Comparisons between the current densities generated from artificial and from real electrode models reveal that current flow prediction in tDCS can be further improved with precise information of electrode location. While the real electrode models depict actual location of electrodes at the time of stimulation, most clinical tDCS studies typically perform stimulation outside of the MRI scanner and do not have electrode imaging data. However, documentation such as picture/photograph, diagram, or 3D scans (Indahlastari et al., 2019a) can be used to inform model frameworks when using automated current flow software. This can be accomplished by selecting a more accurate electrode location from the virtual 10–20 EEG cap that matches the actual location at the time of stimulation, instead of merely using the ideal montage placement intended for the study. For instance, this study was planned to use F3-F4 montage. Based on the real electrode information, selecting cathode electrode at F5 location instead of F3 for participant 3 during an automatic modeling step might be more appropriate to mimic actual electrode placement. Therefore, pairing the utilization of end-to-end current flow modeling software with electrode documentation can increase both the robustness and accuracy of generated current flow models. The accuracy of these models is essential for visualizing the effects of stimulation on an individual’s brain as even small variations in electrode location can result in significant differences in current distribution downstream. As tDCS is utilized in older populations to target specific brain regions associated with cognitive decline, precise models combined with refined electrode placement methods can assist in achieving optimal current values for these targeted regions.

While a significant difference was found in computed current densities between the artificial and real electrode models, the extent of the current density differences varied across sub-cortical locations. Percent difference of current densities in the ROIs, which represented the difference between the generated current densities using artificial versus real electrode models, showed differences of up to 38% (Figure 5D). The electrode montage F3-F4 typically targets the dorsolateral prefrontal cortex (DLPFC) (Nord et al., 2013; Tremblay et al., 2014; Dedoncker et al., 2016) to enhance executive functions, including working memory improvement and speed processing. Both the superior frontal gyrus (Brodmann Area 9) and the middle frontal gyrus (Brodmann Areas 9 and 46) anatomically represent the DLPFC regions (Mai and Paxinos, 2012; Cieslik et al., 2013). Moreover, the F3-F4 montage has also targeted the inferior frontal gyrus to improve response inhibition, migraine, and pain control (DaSilva et al., 2015; Stramaccia et al., 2015; Thunberg et al., 2020). Regarding the target brain regions, smaller differences in current density were noted in regions closer to the electrode location, such as the superior frontal gyrus, the middle frontal gyrus, and the inferior frontal gyrus. In contrast, for non-target regions, larger differences and a more pronounced positive correlation were observed in regions farther from the electrodes, such as the temporal lobe. Therefore, using artificial electrode models with location information solely sourced from the study montage should be taken with caution when analyzing subcortical regions further away from electrode locations. In addition, the superior frontal gyrus (SFG) displayed a significant negative correlation, while the other ROIs showed positive correlations. Although the SFG correlation was negative, it had a relatively shallow gradient. We suspect this could be due to variations in individual electrode placements, particularly the asymmetrical positioning of electrodes around the SFG ROI. This resulted in a unique, mild negative correlation compared to other ROIs. This observation suggests that the other ROIs were less affected by electrode asymmetry. Hence, future studies investigating the distance between the ROI and the stimulation site as a potential mediator might offer more profound insights into these variations.

Normalized electrode contact areas observed in the real models implied that actual contact areas deviated from the planned electrode size. Our segmented electrodes did not align with the presumed 35 cm^2^ planned electrode surface area, yet they reflected the actual stimulation condition. Certain obstacles, such as hair or the placement of the MR head coil, can impede accurate electrode positioning in real-world scenarios and affect resulted contact areas. This discrepancy underscored the motivation for our study to carefully documenting electrode placement information at the time of stimulation. Unfortunately, when creating models based on clinical study, it is common in tDCS modeling to rely solely on the ideal electrode position set at the planned location, without cross-referencing them with the actual/real electrode locations. Therefore, it is crucial that a modeling study corresponds closely with its clinical counterpart, taking meticulous care to ensure electrode positions are as precise as possible. For instance, our previously published study comparing modeling and functional connectivity from the same data utilized the segmented electrodes in the model sourced from the MR data instead of the ideal location of the planned electrode montage (Indahlastari et al., 2021a). Nevertheless, when applying tDCS, the researchers should ensure the electrodes are placed in the planned locations as accurately and consistently as possible, as electrode placement discrepancy beyond 1 cm from the intended location can reduce the accuracy of generated electric fields (Opitz et al., 2018).

### Model limitation and future direction

We acknowledge several limitations within this study that might affect our reported findings. The accuracy of manually segmented real electrode models, such as electrode pads and paste, were limited by the quality (e.g., image resolution and contrast) of participants’ T1-weighted images. Therefore, there may be imperfections, such as overestimation or underestimation of electrode edges and shape at the interface of electrode-scalp boundary, resulting in inconsistent electrode size and affecting their modeled contact areas. Further, the size of the artificial electrode gel was not adjusted to match the presumed actual paste thickness of 5 mm; instead, it retained the assigned electrode pad size thickness of 3 mm. Modifying this was not feasible without explicitly altering the default ROAST code. However, we compared current density volumes for the whole head (J_whole–head_) and the brain (J_brain_) using electrode thicknesses of 3 mm versus 5 mm. The results were very similar (average SSIM = 0.9998 Dice: 1 for J_brain_ and SSIM = 0.9987 and Dice = 0.9999 for J_whole–head_) as shown in Supplementary Table 1. Additionally, the electrodes segmented in the real models might have a different thickness compared to those in the artificial models. The actual electrodes were segmented based on the information from the T1 data. While physical measurements were performed to obtain F3 and F4 location in actual stimulation, the final measurements of the electrode information, such as electrode contact area and distance between the two electrodes, were not physically recorded during the live tDCS sessions. The lack of physical measurements of real electrode final location prevented us from directly checking our real electrode segmentation quality. Therefore, while real electrode location depicted a more accurate representation of the electrode positioning during the stimulation session, its accuracy can be improved with additional documentation. Further, this comparison study was conducted across a small sample of sixteen older adults. The average of differences in median current density between the two electrode types may change with an increased sample size and/or different population types. Therefore, the results in this study will need to be verified in a larger population.

At present, applications of tDCS employ a fixed dosing approach, wherein the same stimulation parameters (including electrode location) are prescribed across all participants within a single study. While tDCS outcomes are promising, there is variability in the success of treatment among recipients, even when using a fixed dosing approach. This variability may be attributed, at least partly, to the inter-individual variability of brain atrophy that occurs with aging and to the human error that occurs when placing the electrodes. Computational models are widely used to predict behavioral responses to stimulation, and they typically use artificially created electrodes to represent intended montages. However, the final electrode location used during stimulation might deviate from the planned locations in intended montages, leading to inaccurate field measurement generation. Based on the findings of this study, rigorous documentation of electrode locations is eminent to producing more accurate field measures (e.g., current density, electric field). This documentation can further aid in selecting electrode locations that are more precise when utilizing automated current flow modeling software and thus produce a more robust prediction.

## Conclusion

This is the first study of its kind to evaluate the accuracy of computational models demonstrating the delivery of current in older individuals by using the manually segmented electrode models as a baseline for comparison. We found a significant difference (*p* < 0.001) between the current density values generated by simulations using artificially created electrode models and those using the manually segmented electrodes. The findings of this study strongly suggest that rigorous documentation of electrodes (e.g., location, separation distance, size, contact area, etc.) at the time of stimulation provides crucial information for generating a more accurate prediction of tDCS electric fields. These findings further inform future research practices in tDCS or related fields, particularly those that involve computational models to predict clinical outcomes of non-invasive brain stimulation such as tDCS.

## Data availability statement

The datasets presented in this article are not readily available because collected data are managed under the data-sharing agreement established with NIA and the parent clinical trial. All data requests will require submission of data use statement, authorship, and analytic plan for review by the study PI. Requests to access the datasets should be directed to AW, ajwoods@phhp.ufl.edu.

## Ethics statement

The studies involving humans were approved by the Institutional Review Boards at the University of Florida. The studies were conducted in accordance with the local legislation and institutional requirements. The participants provided their written informed consent to participate in this study.

## Author contributions

AI: Conceptualization, Data curation, Formal analysis, Investigation, Methodology, Project administration, Software, Validation, Visualization, Writing—original draft, Writing—review and editing. AD: Data curation, Formal analysis, Investigation, Software, Visualization, Writing—original draft, Writing—review and editing. SP: Data curation, Formal analysis, Investigation, Software, Visualization, Writing—review and editing. JK: Data curation, Formal analysis, Software, Visualization, Writing – review and editing. SS: Formal analysis, Visualization, Writing—review and editing. AA: Formal analysis, Visualization, Writing – review and editing. AW: Conceptualization, Funding acquisition, Investigation, Resources, Supervision, Validation, Writing—review and editing.
