# Supplementary material for: Impact of electrode selection on modeling tDCS in the aging brain
Source: Front Hum Neurosci. 2023 Nov 24;17:1274114. doi: 10.3389/fnhum.2023.1274114 (PMC10704166; doi:10.3389/fnhum.2023.1274114)
Supplement: Supplementary file 1 [file Data_Sheet_1.PDF]

APPENDIX A

Electrode imprints

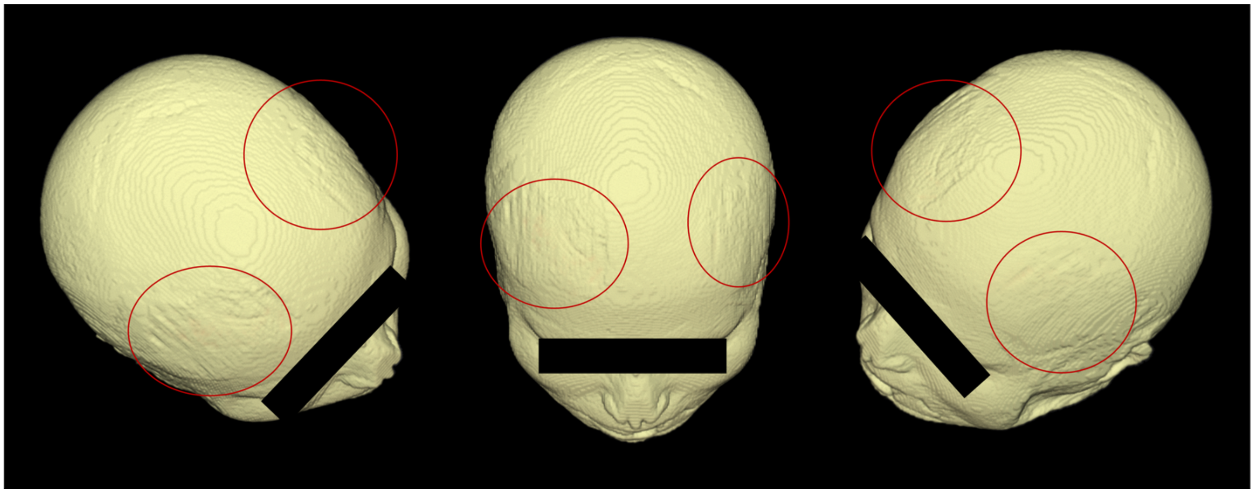

Figure S1. Electrode imprints on a participant's skin/scalp in one participant. Imprinted electrodes on the skin/scalp are circled in red, suggesting that the MR head coil might have applied slight pressure to the skin beneath them.

APPENDIX B

T1-weighted and electrode images

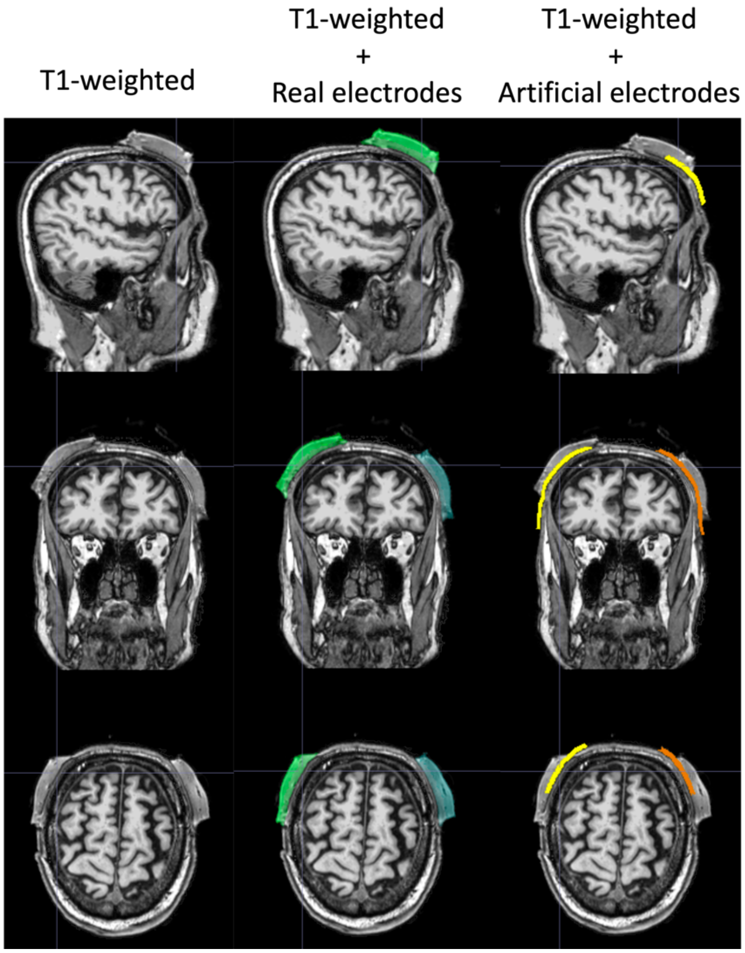

Figure S2. T1-weighted images with and without real and artificial electrodes for one participant. Real electrodes are depicted in green (cathode) and teal (anode) color, while artificial electrodes are depicted in yellow (cathode) and orange (anode) color.

APPENDIX C

Electrode distance definition

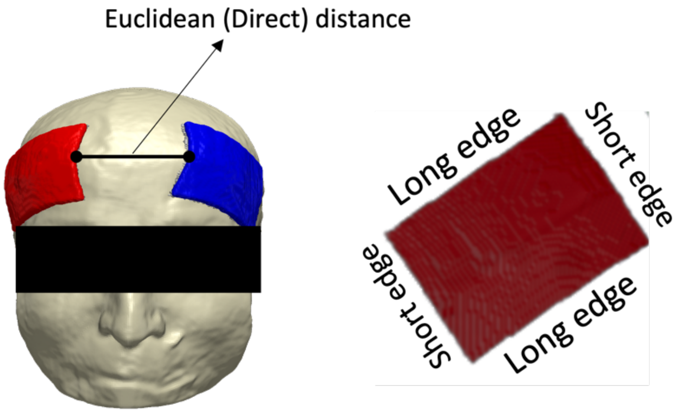

Figure S3. Definition of electrode separation distance. The Euclidean (direct) distance is calculated between the midpoints of the short edges of the electrode pair, as illustrated above.

APPENDIX D

Electrode thickness comparison

SSIM and Dice score were computed between current density volumes resulted from using electrode dimension of 70x50x3 mm versus 70x50x5 mm. In the SSIM metric, where x and y are both current density volumes with 3mm and 5mm thickness, respectively. The variable σ_x^2 is the variance of x, σ_y^2 is the variance of y, σ_{xy} is the covariance of x and y, $c_1 = (k_1 L)^2$ and $c_2 = (k_2 L)^2$ are two variables that stabilize the division,L is the dynamic range of the pixel-values k_1 and k_2 are constants with defaults of 0.01 and 0.03, respectively. In the dice equation, the variable Y was assigned current density with the 3mm thickness and $\widehat{Y}$ was assigned current density the 5 mm thickness.

Table S1. Calculation of SSIM and Dice score for current density volumes derived from electrode thickness of 3 mm vs. 5 mm. The SSIM metric was computed by the following equation: $SSIM(x,y) = \frac{(2\mu_x\mu_y+c_1)(2\sigma_{xy}+c_2)}{(\mu_x^2+\mu_y^2+c_1)(\sigma_x^2+\sigma_y^2+c_2)}$ [1]. The Dice score was computed by the following equation: $Dice = \frac{2|Y \cap \widehat{Y}|}{|Y|+|\widehat{Y}|}$ [2]. The maximum SSIM and Dice score value of 1 indicates that the two volumes are identical. The average SSIM and Dice for J_{brain} are 0.9998 and 1, respectively. The average SSIM and Dice for $J_{whole\text{-}head}$ are 0.9987 and 0.999, respectively.

Participant	J_{brain} SSIM	J_{brain} Dice	$J_{whole\text{-}head}$ SSIM	$J_{whole\text{-}head}$ Dice
1	0.999752558	1	0.998898075	0.999999768
2	0.999760686	1	0.998601803	0.999999583
3	0.999730186	1	0.998786071	0.999998903
4	0.999739108	1	0.998657175	0.999999484
5	0.999712671	1	0.998882031	0.999999124
6	0.999723306	1	0.998843203	0.999999776
7	0.999729247	1	0.998482768	0.999999875
8	0.999842293	1	0.998736798	0.999999299
9	0.999703352	1	0.998784863	0.999998976
10	0.999802759	1	0.998939658	0.999999899
11	0.999780463	1	0.998783972	0.99999989
12	0.999709412	1	0.998187867	0.999999807
13	0.999790908	1	0.998994017	0.999999779
14	0.999822297	1	0.998544841	0.999999336
15	0.999764979	1	0.998762229	0.999999269
16	0.999786403	1	0.998980989	0.999999752

[1] Zhou Wang, A. C. Bovik, H. R. Sheikh and E. P. Simoncelli, "Image quality assessment: from error visibility to structural similarity," in IEEE Transactions on Image Processing, vol. 13, no. 4, pp. 600-612, April 2004, doi: 10.1109/TIP.2003.819861.
[2] Dice, Lee R. “Measures of the Amount of Ecologic Association Between Species.” Ecology, vol. 26, no. 3, 1945, pp. 297–302. JSTOR, https://doi.org/10.2307/1932409.
